# Structural Abnormalities of Spermatozoa in Triploid Gynogenetic Crucian Carp (*Carassius auratus*)

**DOI:** 10.3389/fgene.2021.783014

**Published:** 2021-11-15

**Authors:** Wangchao He, Yu Sun, Jiaxu Qiang, Xinyue Luo, Hui Zhang, Conghui Yang, Kaikun Luo, Rurong Zhao, Qinbo Qin, Chun Zhang, Shaojun Liu

**Affiliations:** State Key Laboratory of Developmental Biology of Freshwater Fish, College of Life Sciences, Engineering Research Center of Polyploid Fish Reproduction and Breeding of the State Education Ministry, Hunan Normal University, Changsha, China

**Keywords:** triploid, spermatozoa, centriole, midpiece, gynogenesis

## Abstract

The spermatozoa of triploid gynogenetic crucian carp (*Carassius auratus*) (3nDTCC) possess a spermatogenesis process with a normal genetic background. However, the genetic materials of these spermatozoa do not completely inherit gynogenetic progeny in general. Understanding the intrinsic mechanism may be helpful for developing breeding strategies of gynogenetic fishes. In this study, the spermatozoa ultrastructure was systematically studied in diploid red crucian carp and 3nDTCC to demonstrate their cytological structural differences. In addition, the artificial breeding tests of 3nDTCC(♀) with different ploidy spermatozoa were performed to verify the contributions of genetic materials from 3nDTCC spermatozoa to the gynogenesis progeny. Furthermore, the mRNA expression of centriole-related genes (i.e., *cep57*, *cetn1*, *rootletin*, and *nek2*) involved in spermatozoa packaging was also determined by quantitative real-time PCR (qPCR) to illustrate the molecular expression characteristics of the spermatozoa packaging process in 3nDTCC. The results reveal the adaptive features of spermatozoa in 3nDTCC, including the loose midpiece structure, abnormal head structure, and abnormal expression of centriole-related genes, which may influence the motility of spermatozoa and make it not involved normally in the genetic composition of the gynogenesis offspring.

## Introduction

Crucian carp (*Carassius auratus* L.,), which belongs to the genus of *Carassius* within the family of Cyprinidae, has a wide geographic distribution in the Eurasian continent. In China, the species has been found in most provinces, and the diverse genetic strains have been identified in different regions. *Carassius auratus gibelio* (*C. gibelio* Bloch) is originated from sympatric progenitor crucian carp (*Carassius auratus*) (Li et al., 2014), which is widely distributed in the Heilongjiang water system and many other natural habitats (Sheng and Wang, 1983; Gui and Zhou, 2010; Zhou et al., 2000). Other crucian carp have been reported in China since 1980s, including the Pengze crucian carp in the waters of Jiangxi province (Yang and Chen, 1992; Chen et al., 1996; Liu et al., 2004; Dong et al., 2004), the high-back crucian carp in the Dianchi lake of Yunnan and its affiliated waters, the Sogu crucian carp in Guangdong province (Yu et al., 1987), the Puan crucian carp in Guizhou province (Yu et al., 1992), and the wild crucian carp in the Dongting lake water system (Xiao et al., 2011; Liu et al., 2012; Qin et al., 2016) and so on. Most crucian carp in the abovementioned different natural waters have two sex ratio groups—bisexual and unisexual. The former always possesses a certain proportion of males, which can reproduce and undergo normal bisexual fertilization. The latter is a natural gynogenetic population, mostly being triploid, with no or a few males. In the natural gynogenetic monosexual population, there were many studies which showed that male individuals have the same genetic background as female individuals (Jiang et al., 1983; Liu et al., 2004), and the nature triploid population is generally propagated by gynogenesis. The spermatozoa of triploids mostly activate only triploid ova, without participating in the genetic makeup of the offspring (Li et al., 2016; Li et al., 2018; Zhu et al., 2018). The cause of spermatozoa with a normal genetic background not involving in the genetic composition of gynogenesis progeny liking in the bisexual fish deserves intensive investigation.

The natural crucian carp (*Carassius auratus* L.,) in the Dongting water system of China has been reported by our group. The population had both diploids (2nDTCC) and triploids (3nDTCC), in which the proportion of triploid (85.0%) was much higher than that in diploid (15.0%), and there was no significant difference in morphological characteristics between diploids and triploids. Among them, the proportion of male in diploid population was about 50.0%, and the proportion of male in triploid population was about 14.3% (Liu et al., 2012). The artificial breeding experiment showed that the male and female 3nDTCC were all fertile and produced triploid offspring when mating with each other (Xiao et al., 2011). The recent research studies have showed that 3nDTCC males presented a relative regular meiotic prophase I with complete conjugate chromosome pairs and finally formed mature sperms (Zhang et al., 2021). The 3nDTCC males are fertile and can produce spermatozoa with “normal morphology” and possess normal swimming ability, but the genetic material of them is rejected by gynogenetic progeny. How can the spermatozoa of 3nDTCC adapt to the propagating mode of gynogenesis? Whether there were structural differences between the spermatozoa of 3nDTCC and the spermatozoa of bisexual reproductive diploid species?

Considering the morphology of the spermatozoa is related to the semen quality; the ultrastructure of spermatozoa in 3nDTCC and 2nRCC was systematically studied to demonstrate their cytological structural differences. In addition, based on detecting the DNA content of semen in 2nDTCC and 3nDTCC by flow cytometric analysis, the artificial breeding tests of 3nDTCC(♀)×2nDTCC(♂) and 3nDTCC(♀)×3nDTCC(♂) were performed to verify if the spermatozoa produced by 3nDTCC do contribute to the gynogenesis progeny, with the ploidy of progeny being detected by the DNA content. Furthermore, the mRNA expression of centriole-related genes (i.e., *cep57*, *cetn1*, *rootletin*, and *nek2*) which were involved in spermatozoa packaging was also determined by quantitative real-time PCR (qPCR) to illustrate the molecular mechanism of the abnormal spermatozoa packaging process in 3nDTCC.

The study will initially reveal the adaptive structural features of spermatozoa in 3nDTCC not involving in the genetic composition of the gynogenesis offspring, which is of great significance in fish evolutionary research and aquatic breeding.

## Materials and Methods

### Experimental Fish

Forty natural individuals, including two male 2nDTCCs and 38 male 3nDTCCs aged 10–11 months, were collected from the State Key Laboratory of Developmental Biology of Freshwater Fish, Hunan Normal University. Moreover mature male 2nRCCs were used as a control to compare the ultrastructures of the testis tissues, and mature male 2nRCCs were used as a control to compare the mRNA expression of centriole-related genes (Xu et al., 2015).

Animal experimenters were certified under a professional training course for laboratory animal practitioners held by the Institute of Experimental Animals, Hunan Province, China. All fish were euthanized using 2-phenoxyethanol (Sigma, United States) before being dissected. The fish were treated humanely following the regulations of the Administration of Affairs Concerning Experimental Animals for the Science and Technology Bureau of China. All applicable institutional and national guidelines for the care and use of animals were followed.

### Electron Microscope Analysis of Sperm

Testis tissue samples of five male 2nRCCs and eight male 3nDTCCs were collected, fixed in 3% glutaraldehyde solution, washed with phosphate buffer, transferred to an osmic acid solution, dehydrated in a graded acetone series, and embedded in Epon812. Ultrathin sections were cut and stained with uranyl acetate and lead citrate. An HT7800 transmission electron microscope (HITACHI, Japan) was used to observe the ultrastructures of the tissues.

### qPCR Analysis

Centriole-related genes (i.e., *cep57*, *cetn1*, *rootletin*, and *nek2*) involved in spermatozoa development were selected for verification and comparison in 2nRCC and 3nDTCC using qPCR. Total RNA was extracted from the remaining testicular tissue and reverse-transcribed into first-strand cDNA using reverse transcriptase (Invitrogen). Real-time PCR was performed using the Prism 7,500 Sequence Detection System (Applied Biosystems, United States) with a PowerUpTM SYBRTM Green Master Mix (Applied Biosystems, United States). Real-time qPCR was performed in triplicates.

The reaction mixture (10 µL) consisted of the following components: 2.5 µL of cDNA (1:4 dilution), 5 µL of PowerUpTM SYBRTM Green Master Mix, 0.5 µL of the specific forward primers, 0.5 µL of the reverse primers, and 1.5 µL of water. The amplification conditions were 50°C for 5 min and 95°C for 10 min, followed by 40 cycles at 95°C for 15 s and 60°C for 45 s. The average threshold cycle (Ct) was calculated for each sample using the 2-ΔΔCt method and normalized according to *β*-actin. Finally, melting curve analysis was performed to validate the specific amplification of the expected products.

### Breeding Tests

The eggs produced by 3nDTCC were fertilized with sperms of male 2nDTCCs and male 3nDTCCs whose ploidy is different. The milt of males was stripped and mixed with the eggs of 3nDTCC (query). After adding water to activate, the fertilized eggs were incubated at 18–20°C in fresh water until hatching, calculating the fertility rate (%), hatching rate (%), and survival rate after 1 month (%). The progeny of each crossing was bred separately, and then, their morphological traits were observed. The ploidy of progeny was detected by the DNA content.

### Measurement of DNA Content

A flow cytometer (Partec GmbH, Germany) was employed to detect the ploidy of sperms in 2nDTCC and 3nDTCC and the ploidy of progeny which originated from different crossing. Spermatic fluid samples were squeezed out from the mature male 2nDTCCs and 3nDTCCs (5–10 µL per fish), or the blood of progeny. The fluid was then treated with a DAPI DNA staining solution for 10–15 min and filtered. The DNA content of the red blood cells was used as a control. Meanwhile, the sperm motility was detected under a microscope.

## Results

### Sperm Motility and DNA Content of Semen in 2nDTCC and 3nDTCC

**FIGURE 1:**
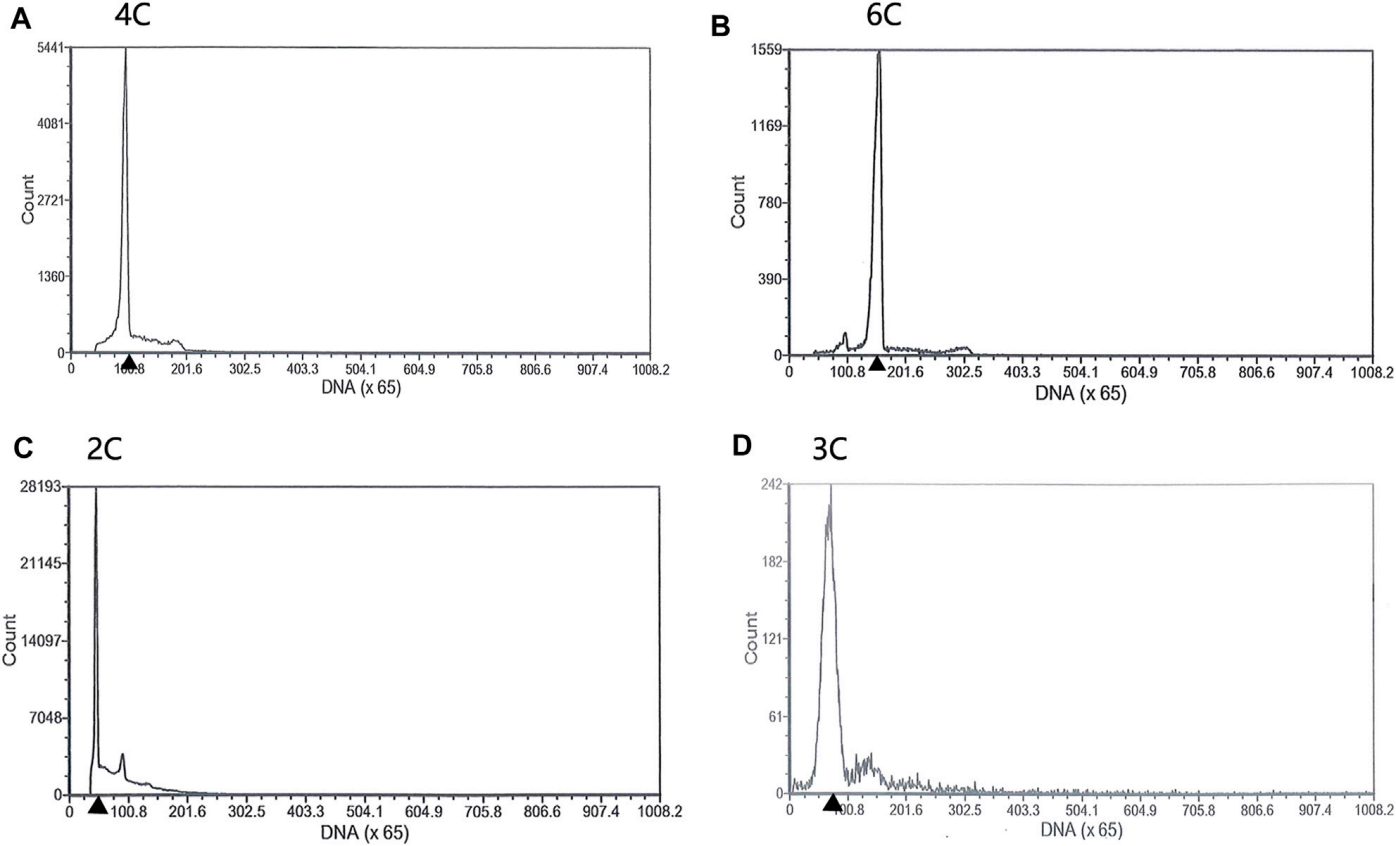
Flow cytometry histograms of semen cells obtained from 2nDTCC and 3nDTCC. The blood cells of 2nDTCC and 3nDTCC were detected as the control. **(A)** Blood cells of 2nDTCC (DNA content, 4C). **(B)** Blood cells of 3nDTCC (DNA content, 6C). **(C)** Semen cells of 2nDTCC (DNA content, 2C). **(D)** Semen cells of 3nDTCC (DNA content, 3C).

Both diploids and triploids showed normal sperm motility by microscopy. The DNA content of the semen being collected from two 2nDTCC males and 10 3nDTCC males were detected by flow cytometry. The blood cells of 2nDTCC and 3nDTCC were used as controls. The results showed that all 2nDTCC samples produced a major peak at approximately 2C DNA content while its blood samples presented 4C DNA content (Figures 1A,C). Meanwhile, all 3nDTCC samples produced a major peak at approximately 3C DNA while its blood samples presented 6C DNA content (Figures 1B,D). Therefore, it is seen that both diploids and triploids produce sperm with halved ploidy. Statistics of the DNA content of semen from 2nDTCC and 3nDTCC were showed in Table 1.

**TABLE 1:** The DNA content of semen collected from 2nDTCC and 3nDTCC.

Samples	Sample number	The DNA content of blood cells	The DNA content of semen	Note
2nDTCC	2	4C/2n	2C/n	Produce sperm with halved ploidy
3nDTCC	10	6C/3n	3C/1.5n	Produce sperm with halved ploidy

### Ultrastructures of Testes From 2nRCC and 3nDTCC

**FIGURE 2:**
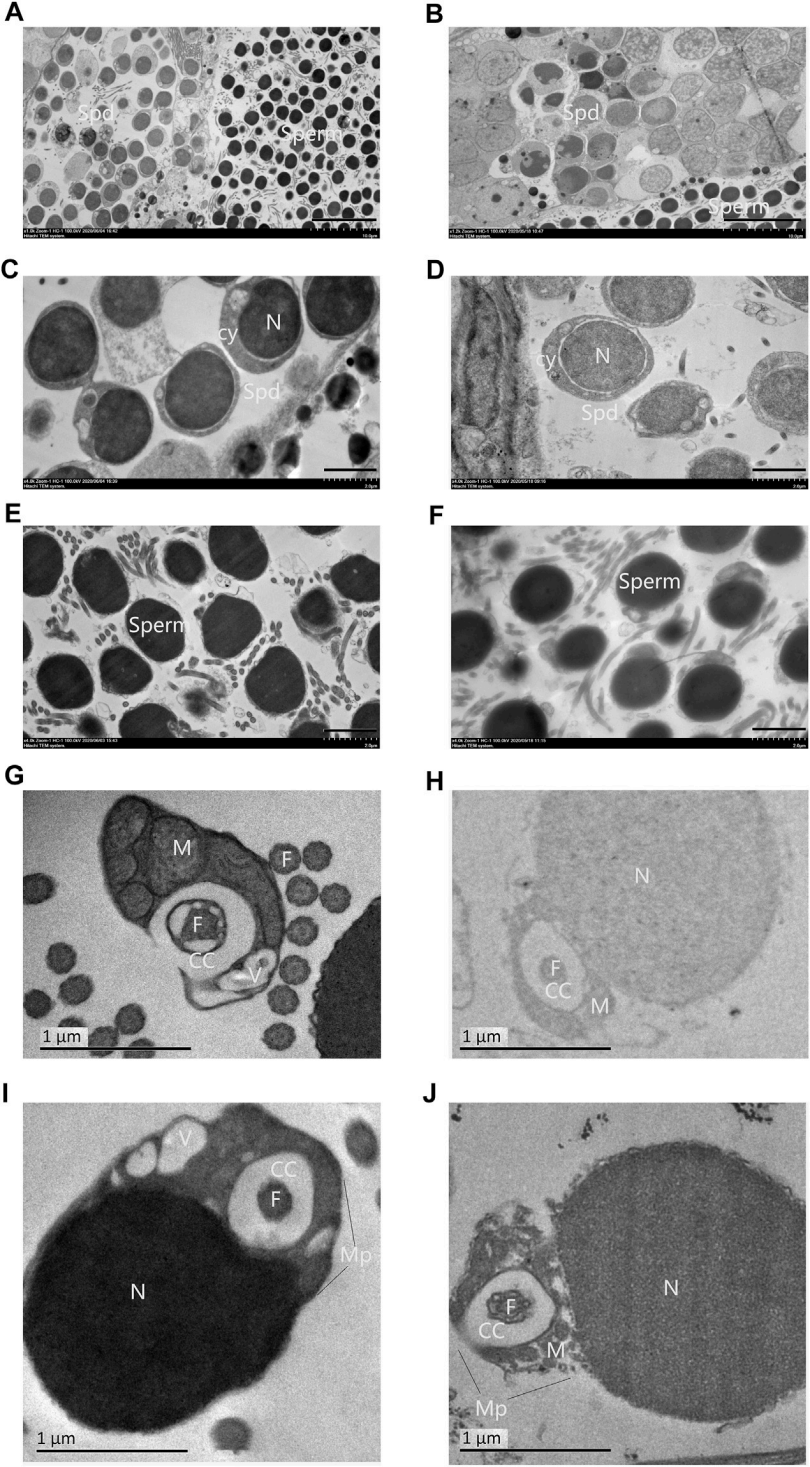
Ultrastructures of the testes from 2nRCC and 3nDTCC. The ultrastructures of testes from 2nRCC were showed in **(A)**, in which spermatids were packed with higher nucleo-cytoplasmic ratios **(C)**, the spermatozoa heads are concentrated densely **(E)**, and the midpiece organized tightly **(G, I)**. The ultrastructures of testes from 3nDTCC were showed in **(B)**, in which spermatids were packed with lightly stained nuclei **(D)**, the spermatozoa heads are concentrated densely **(F)**, and the midpiece organized loosely **(H, J)**. Spd, spermatid; Cy, cytoplasm; N, nucleus; V, vesicle; Cc, cytoplasmatic channel; F, flagellum (centriole); Mp, midpiece; and M, mitochondria. Bar in A, B is 10 μm; Bar in C-F is 2 μm; Bar in G-J is 1 µm.

Spermatozoa of 2nRCC typically consists of a primitive head without acrosome, a midpiece with several mitochondria, a centriolar complex (proximal and distal centriole), and one flagellum. A holistic view of testes for TEM in 2nRCC and 3nDTCC showed alternate distribution of the spermatid region and mature spermatozoa region (Figures 2A,B). Here, spermatids were packed with higher nucleo-cytoplasmic ratios and the nucleus concentrated densely, with the staining contrast between the nucleus and the cytoplasm being evident in 2nRCC (Figure 2C), while the nucleus concentrated lightly in 3nDTCC (Figure 2D). The overall structure of the spermatozoa heads in 2nRCC and 3nDTCC is similar, being mostly composed of dense and slightly granular material, which appeared to be fairly homogeneous (Figures 2E,F). It is worth mentioning that significant differences are seen in the midpiece region of spermatozoa, which were magnified to see mitochondria and vesicles being tightly organized around the centriolar complex in 2nRCC (Figures 2G,I), while that is loose in 3nDTCC (Figures 2H,J).

### Abnormal Structure in 3nDTCC Spermatozoa

**FIGURE 3:**
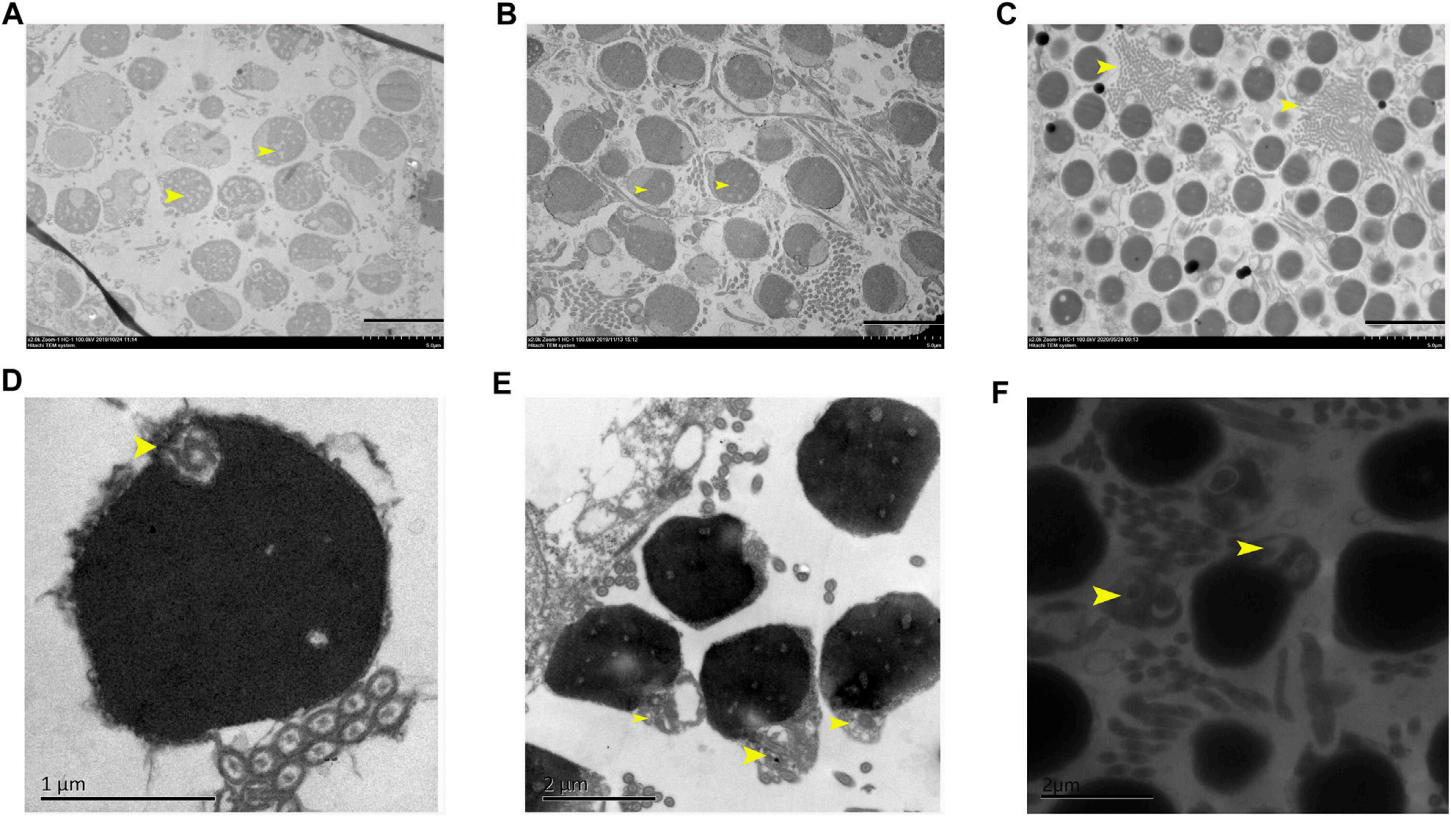
Abnormal structural features in 3nDTCC sperm. **(A)** The nuclei are heavily vacuolated; **(B)** the head shape is irregular; **(C)** a large number of spermatozoa axial cross sections are concentrated; small centriolar complex **(D)**, abnormal link of head with flagella **(E)** and a spermatozoa with two flagella **(F)** are magnified. Bar in A, B, C is 5 μm; bar in D is 1 μm; bar in E and F is 2 µm.

Overall spermatozoa morphological quality is not good in all samples, in which the normal mature spermatozoa region and abnormal spermatozoa region alternately distributed. Some abnormal spermatozoa showed nuclei being heavily vacuolated (Figure 3A), some showed the head shape being irregular (Figure 3B), and some regions showed a large number of spermatozoa axial cross sections being concentrated (Figure 3C). Furthermore, a small centriolar complex (Figure 3D), abnormal link of head with flagellum (Figure 3E), and two flagella with a communal midpiece, but separate cytoplasmic channels (Figure 3F) were found.

### qPCR Validation

**FIGURE 4:**
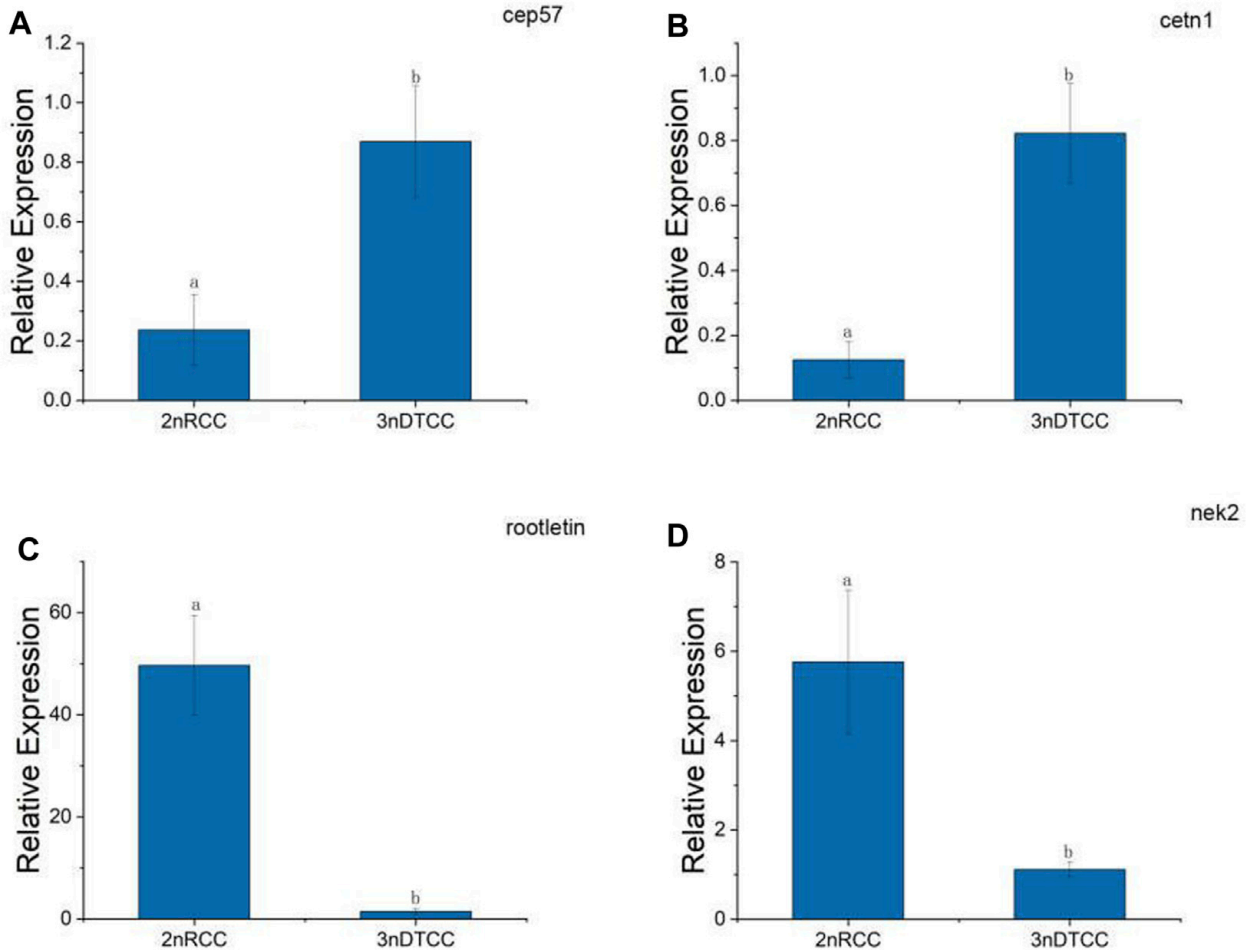
Real-time PCR analysis for *cep57*, *cetn1*, *rootletin*, and *nek2* in 2nRCC and 3nDTCC. **(A)** *cep57*, centrosomal protein of 57 kDa; **(B)** *cetn1,* centrin 1; **(C)** *rootletin,* **(D)** *nek2,* NIMA-related kinase2. The different lowercase letters in each panel indicate significant differences at *p* < 0.05 (mean ± SD of relative expression; n = 9 for each group).

By the result of electron microscopy, we speculate that the centriole packing process of 3nDTCC spermatozoa is abnormal compared to that in bisexual diploid fish. So we further investigate the expression differences of the relevant genes. A typical centriole contains a rich variety of centriole proteins that assemble their various substructures (Avidor and Fishman, 2019). A centrosomal protein of 57 kDa (CEP 57) is required for pericentriolar material (PCM) organization that regulates centriole engagement (Watanabe et al., 2019). Centrin 1 (CETN1) forms the centriole cartwheel and CETN1 proteins are conserved centriolar proteins that fill the lumen of the mature centriole (Avidor-Reiss et al., 2020). CETN1 is the only member that is specifically enriched in spermatozoa cells. Rootletin and NIMA-related kinase2 (NEK2) are centriole proteins being required for flagellum formation and linking the head to the tail (Avidor-Reiss et al., 2020). We chose the four representative genes and performed qPCR on biological replication in triplicates. qPCR showed that the expression levels of *cep57* gene and *cetn1* gene are higher in 3nDTCC than in 2nRCC, and the expression levels of *rootletin* gene and *nek2* gene are lower in 3nDTCC than in 2nRCC (Figure 4).

### Breeding Tests of Sperm From 2nDTCC and 3nDTCC

**FIGURE 5:**
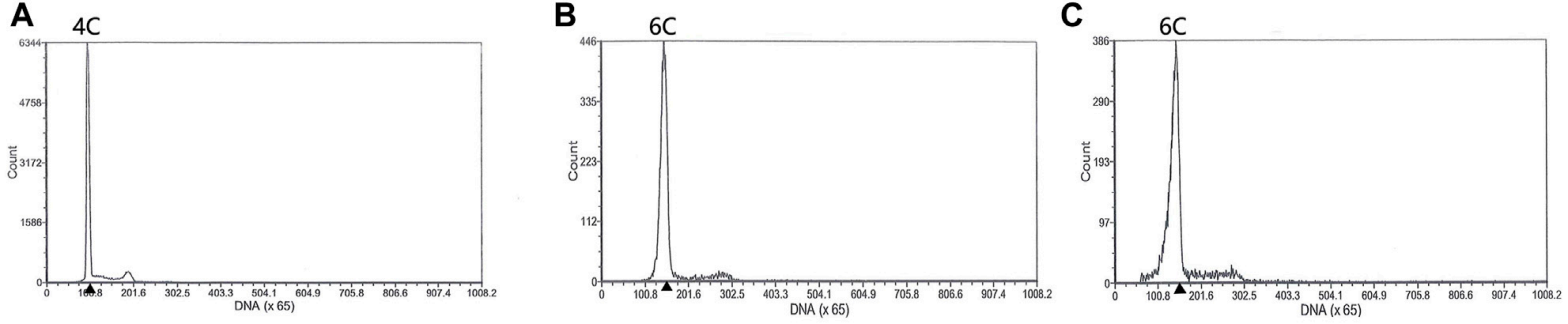
Flow cytometry histograms of blood cells obtained from progeny of different crossing progeny. The blood cells of RCC were detected as the control. **(A)** Blood cells of RCC (DNA content, 4C). **(B)** Blood cells of the progeny in 3nDTCC(♀)×2nDTCC(♂) (DNA content, 6C). **(C)** Blood cells of the progeny in 3nDTCC(♀)×3nDTCC(♂) (DNA content, 6C).

In order to explore the contribution of different ploidy sperms to gynogenesis fish, the different crossings were carried out, including three groups of 3nDTCC(♀)×2nDTCC(♂) and three groups of 3nDTCC(♀)×3nDTCC(♂). In 3nDTCC(♀)×2nDTCC(♂) breeding tests, all the crossings had a high fertilization rate (>80.0%), but a low hatching rate (28.0–46.0%) and survival rate after 1 month (<12.0%). The morphological traits of progeny were similar to those of the female 3nDTCC, and the ploidy of progeny was triploid (Figure 5). The results indicated that the haploid spermatozoa produced by 2nDTCC only activated the eggs’ development, the chromosomes of which did not normally contribute to the gynogenesis progeny as that in bisexual diploid fish. In 3nDTCC(♀)×3nDTCC(♂) breeding tests, the similar results indicated that the 1.5n spermatozoa produced by 3nDTCC also did not normally participate in the gynogenesis progeny as that in bisexual diploid fish (Table 2).

**TABLE 2:** Breeding tests of semen from 2nDTCC and 3nDTCC crossing with the eggs of 3nDTCC.

Breeding group		Fertility rate (%)	Hatching rate (%)	Survival rate after 1 month (%)	DNA content of progeny (blood cells)
3nDTCC(♀)×2nDTCC(♂)	Group-A	80.3	41.2	10.6	6C/3n
	Group-B	85.2	35.5	8.0	6C/3n
	Group-C	80.2	28.0	6.0	6C/3n
3nDTCC(♀)×3nDTCC(♂)	Group-A	80.1	45.6	11.3	6C/3n
	Group-B	82.1	30.3	9.0	6C/3n
	Group-C	81.6	40.0	11.0	6C/3n

## Discussion

Gynogenesis fish often are not dependent on the involvement of sperm genetic material, in which the sperms are required to stimulate the eggs to initiate embryogenesis using only maternal genetic information (Gui and Zhou 2010). The introgression of paternal DNA might play an important role in the creation of genetic diversity in gynogenetic fish, for example, extra microchromosomes in males are closely related to male determination in gynogenetic gibel carp (Li et al., 2016; Zhao et al., 2021; Ding et al., 2021), and the insertion of paternal DNA was also reported in gynogenetic blunt snout bream (Gong et al., 2019).

3nDTCC is generally propagated by gynogenesis. The spermatozoa of the 3nDTCC mostly activate triploid ova and do not participate in the genetic makeup of offspring (Xiao et al., 2011). Furthermore, 3nDTCC possessed a complete spermatogenesis process to produce viable 1.5n sperm (Zhang et al., 2021), and the breeding test of 3nDTCC (♀) ×3nDTCC (♂) showed that the 1.5n spermatozoa only activated the eggs, not involved in the genetic composition of the offspring or only inserted a few of the paternal DNA fragments, which were speculated by the progeny were triploid and all-female. Do the spermatozoa of 3nDTCC possess the complete structure as that in bisexual reproductive diploid fish?

The typical fish spermatozoa consist of a primitive head without acrosome, a midpiece with several mitochondria, a centriolar complex (proximal and distal centriole), and one flagellum (Psenicka et al., 2009; Zhu et al., 2021). The basic task of the spermatozoon head is to transfer the genetic material localized in the nucleoplasma to the egg. The optimal shape and size of the spermatozoon head is a prerequisite for good penetration of the spermatozoon throughout the micropyle. In the study, ultrastructures of the testes have revealed some malformation in the spermatozoa formation process of 3nDTCC, while spermatids were packed slightly stained nuclei, some spermatozoa heads are concentrated lightly, some heavily vacuolated head, irregular shape head, and abnormal flagella are easily detected. Most important of all, the midpiece structure of 3nDTCC organized looser than that in 2nRCC. Expression validation of centriole-related genes indicate that the expression levels of *cep57* gene and *cetn1* gene, which are required for pericentriolar material (PCM) organization and the centriole cartwheel, are higher in 3nDTCC than in 2nRCC, and the expression levels of *rootletin* gene and *nek2* gene, which are required for flagellum formation and linking the head to the tail, are lower in 3nDTCC than in 2nRCC. As the unnormal expression of centriole proteins, including up adjustment and down adjustment, directly affected recruiting the related proteins, which combined to affect the organization of the structure of PCM and linker (Watanabe et al., 2019; Avidor-Reiss et al., 2020), with both the PCM and linker being the important components of the midpiece, we conclude that the differential expression of centriole-related genes of 3nDTCC must be related to the malformation phenomenon in the midpiece of spermatozoa. The differential expression of centriole-related genes covering the molecular mechanisms needs further investigation.

As the midpiece has important functions in connecting the head to the flagellum (centriolar complex) and providing energy for sperm movement of the spermatozoon (mitochondria), and it has an influence on the movement and structure of the flagellum (Psenicka et al., 2009), we speculate that the motility of spermatozoa in 3nDTCC was different from that in bisexual diploid fish. As the lack of acrosome is compensated by the presence of a micropyle in the egg for penetration of the spermatozoon in fishes (Psenicka et al., 2006), we speculate that whether the nucleus of the spermatozoon that can go into the egg would be related to the motility of spermatozoa tails, which might push the sperm nucleus go into the egg. Thus, the dynamics of sperm tails is an important point which is worth further concerning in fish breeding. The breeding test of all the crossings having a high fertilization rate (>80.0%), but low hatching rate (28.0–46.0%) and survival rate after 1 month (<12.0%), might partly attribute to low incubation water temperature (18–20°C), which has affected the late development of the gynogenesis embryos. It is also possible that poor quality sperm leads to the low survival of gynogenesis offspring.

The malformation phenomenon in the spermatozoa formation process of 3nDTCC might be an adapted feature for the propagating mode of gynogenesis, which make the 3nDTCC spermatozoa maintain the basic structure and movement ability, to reach the egg surface, and to initiate egg development, but not enough ability to break through the egg surface obstacles and to transfer the genetic material into the fertilized egg. Of course, the triploid egg also has its special shielding system, the breeding test of 3nDTCC(♀)×2nDTCC(♂) showed that the haploid genome of bisexual spermatozoa were also found to be not able to incorporate into the 3nDTCC genome. Further research is needed on how the triploid eggs work and how the initiation process achieves the introgression of paternal DNA.

The spermatozoa of gynogenesis fish only start the egg development in the reproduction process. Its adaptive structure related to spermatozoa vitality and spermatozoa entry is worthy of deep understanding. It is not only of great significance in the aspect of biological evolution research, but also provides important theoretical guidance for the inactivation process of artificial gynogenetic induction.

## Data Availability

The original contributions presented in the study are included in the article/Supplementary Material; further inquiries can be directed to the corresponding authors.
